# SHIV-1157i and passaged progeny viruses encoding R5 HIV-1 clade C env cause AIDS in rhesus monkeys

**DOI:** 10.1186/1742-4690-5-94

**Published:** 2008-10-17

**Authors:** Michael Humbert, Robert A Rasmussen, Ruijiang Song, Helena Ong, Prachi Sharma, Agnès L Chenine, Victor G Kramer, Nagadenahalli B Siddappa, Weidong Xu, James G Else, Francis J Novembre, Elizabeth Strobert, Shawn P O'Neil, Ruth M Ruprecht

**Affiliations:** 1 Dana-Farber Cancer Institute, 44 Binney Street, Boston, MA 02115, USA; 2 Harvard Medical School, 25 Shattuck Street, Boston, MA 02115, USA; 3 Yerkes National Primate Research Center, Emory University, 954 Gatewood Road NE, Atlanta, GA, 30329, USA; 4 New England Primate Research Center, PO Box 9102, Southborough, MA 01772, USA

## Abstract

**Background:**

Infection of nonhuman primates with simian immunodeficiency virus (SIV) or chimeric simian-human immunodeficiency virus (SHIV) strains is widely used to study lentiviral pathogenesis, antiviral immunity and the efficacy of AIDS vaccine candidates. SHIV challenges allow assessment of anti-HIV-1 envelope responses in primates. As such, SHIVs should mimic natural HIV-1 infection in humans and, to address the pandemic, encode HIV-1 Env components representing major viral subtypes worldwide.

**Results:**

We have developed a panel of clade C R5-tropic SHIVs based upon *env *of a Zambian pediatric isolate of HIV-1 clade C, the world's most prevalent HIV-1 subtype. The parental infectious proviral clone, SHIV-1157i, was rapidly passaged through five rhesus monkeys. After AIDS developed in the first animal at week 123 post-inoculation, infected blood was infused into a sixth monkey. Virus reisolated at this late stage was still exclusively R5 tropic and mucosally transmissible. Here we describe the long-term follow-up of this initial cohort of six monkeys. Two have remained non-progressors, whereas the other four gradually progressed to AIDS within 123–270 weeks post-exposure. Two progressors succumbed to opportunistic infections, including a case of SV40 encephalitis.

**Conclusion:**

These data document the disease progression induced by the first mucosally transmissible, pathogenic R5 non-clade B SHIV and suggest that SHIV-1157i-derived viruses, including the late-stage, highly replication-competent SHIV-1157ipd3N4 previously described (Song et al., 2006), display biological characteristics that mirror those of HIV-1 clade C and support their expanded use for AIDS vaccine studies in nonhuman primates.

## Background

Animal models of viral diseases have contributed significantly towards our understanding of virus life cycles, routes of transmission and pathologic sequelae following infection. In the case of HIV, macaque models are used to mimic HIV transmission and disease progression in humans, using either simian immunodeficiency virus (SIV) or chimeric simian-human immunodeficiency virus (SHIV) strains that can be tracked prospectively by markers such as plasma viremia levels and loss of peripheral blood CD4^+ ^T cells. Nonhuman primate models of HIV infection are also used to study the efficacy of candidate vaccines and to evaluate innate and adaptive immune responses to the virus. However, to obtain biologically relevant results from animal models, the challenge viruses used should mirror naturally occurring HIV infection in humans and therefore should: 1) be highly replication competent, 2) be mucosally transmissible and use the CCR5 coreceptor for target cell entry, as 90% of all HIV transmissions occur mucosally and almost always involve R5 viruses [1-7], 3) induce disease in a pattern of acute and chronic phases approximating natural disease progression in HIV-infected patients, and 4) cause a relatively slow onset of AIDS.

We developed a clade C SHIV (SHIV-C), termed SHIV-1157i, which encodes an envelope derived from a Zambian infant recently infected with clade C HIV (HIV-C) [8]. SHIV-1157i was then adapted to rhesus monkeys by rapid animal-to-animal passage. Here we describe clinical data from the initial cohort of six animals exposed to the virus during the course of serial viral passage. We show that infection of macaques with either SHIV-1157i or with passaged virus leads to depletion of both memory and total CD4^+ ^T cells, resulting in AIDS and multiple opportunistic infections in some monkeys. Importantly, these hallmarks of primate immunodeficiency virus virulence arose gradually, reflecting the disease progression rate seen in HIV-infected humans.

## Methods

### Virus isolate

The origin, cloning and nomenclature of SHIV-1157i, SHIV-1157ipd and SHIV-1157ipd3N4 is described elsewhere [8]. Briefly, SHIV-1157i is an infectious molecular clone, SHIV-1157ip designates the passaged virus, a biological isolate derived from monkey RKl-8 (passage 4).

### Animals and animal care

Six rhesus monkeys (*Macaca mulatta*) of Indian origin were used for this study. The first recipient was inoculated i.v. with 6 ml cell-free supernatant from 293T cells transfected with the infectious molecular clone, SHIV-1157i. Plasma vRNA loads were measured weekly; if week 1 loads were ≥ 10^4 ^copies/ml, 1 ml of infected blood was transfused at week 2 post-inoculation to the next animal. In each case, peak viremia occurred at week 2. Monkey RBg-9 was inoculated i.v. one month after onset of AIDS in RPn-8 (week 123 p.i.) by transfusing 10 ml of blood. All animals were kept according to National Institutes of Health guidelines on the care and use of laboratory animals at the Yerkes National Primate Research Center (Emory University, Atlanta, GA). The facility is fully accredited by the Association for Assessment and Accreditation of Laboratory Animal Care International. All experiments were approved by the Animal Care and Use Committees of the Yerkes National Primate Research Center and the Dana-Farber Cancer Institute.

### Plasma vRNA loads

RNA was isolated from plasma using QiaAmp Viral Mini Kit (Qiagen), and vRNA loads were measured by quantitative reverse transcriptase PCR (RT-PCR) for SIV *gag *sequences [9]. The detection limit was 50 viral RNA copies/ml of plasma.

### Gross pathology

A complete necropsy was performed on RKl-8 and RPn-8 after death or following euthanasia. Representative tissue from brain, heart, lungs, liver, kidneys, spleen, lymph nodes, bone marrow and gastrointestinal tract were collected in 10% neutral buffered formalin.

### Histology

After fixation the tissue samples were sectioned, processed and embedded in paraffin. For histopathological examination, thin sections (5 μm) of paraffin-embedded tissue were stained with hematoxylin and eosin (H&E).

### Immunohistochemistry (IHC)

IHC was performed for simian virus 40 (SV40) and rhesus lymphocryptovirus (LCV), an Epstein-Barr virus (EBV)-related herpesvirus of rhesus monkeys, using a commercial kit (ABC Elite, Vector Laboratories, Burlingame, CA) and monoclonal antibodies (mAbs) that recognize either SV40 large T-antigen (Calbiochem, San Diego, CA) or EBV encoded nuclear antigen 2 (EBNA-2, Leica Microsystems, Bannockburn IL), respectively. Formalin-fixed, paraffin-embedded (FFPE) sections of brain (for SV40) and tongue (for EBNA-2) were deparaffinized in xylene and rehydrated through graded ethanol to distilled water. Endogenous peroxidase activity was blocked by incubation in 3% H_2_O_2_, and antigen retrieval was accomplished by microwaving sections for 20 minutes in citrate buffer (Dako Corp., Carpinteria, CA). Sections were incubated for 30 minutes at room temperature with primary antibodies, and reacted sequentially with appropriate biotinylated secondary antibodies and horseradish peroxidase-conjugated avidin DH. Antigen-antibody complex formation was localized by development in the chromogenic substrate 3, 3'-diaminobenzidine (Dako). Tissue sections were counterstained in Mayer's hematoxylin (Dako), cleared, and coverslipped with permanent mounting medium. Sections of kidney tissue from a rhesus macaque with SV40 nephritis and sections of oropharyngeal mucosa infected with rhesus lymphocryptovirus served as both positive control (when incubated with SV40 or EBNA-2-specific antibodies, respectively) and negative control (when incubated with irrelevant, isotype-matched control immunoglobulins).

### In situ hybridization (ISH) for viral pathogens

ISH was performed to localize SV40 DNA and SIV RNA in FFPE sections of brain. For both reactions, tissue sections were deparaffinized in xylene and rehydrated in graded ethanol to diethyl pyrocarbonate (Sigma-Aldrich, St. Louis, MO) treated water. Endogenous alkaline phosphatase activity was blocked with levamisole (Sigma), and tissue sections were hydrolyzed in HCl (Sigma), digested with proteinase K (Roche Diagnostics, Corp., Indianapolis, IN), and acetylated in acetic anhydride (Sigma). For SV40 detection, sections were covered with a biotinylated DNA probe cocktail that spans the entire genome of SV40 (Enzo Life Sciences Inc., Farmingdale, NY), then heated at 95°C for 5 minutes to denature DNA, and hybridized overnight at 37°C. To detect cells productively infected with SHIV, brain sections were hybridized overnight at 50°C with a digoxigenin-labeled antisense riboprobe that spans the entire genome of the SIVmac239 molecular clone of SIV (Lofstrand Labs, Gaithersburg, MD). For both ISH reactions, tissue sections were washed extensively the following day and bound probe was detected by IHC. Biotinylated SV40 probe was localized with alkaline phosphatase-conjugated streptavidin (Dako) and digoxigenin-labeled SIV probe was detected with alkaline phosphatase-conjugated sheep anti-digoxigenin F(ab) fragments (Roche), in both instances using the chromogen nitroblue tetrazolium/5-bromo-4-chloro-3-indolyl-phosphate (NBT/BCIP) (Roche), and sections were counterstained with nuclear fast red (Vector Labs). For SV40 ISH reactions, sections of kidney from a rhesus macaque with SV40 nephritis served as both positive control (when incubated with SV40 probe) and negative control (when reacted with a biotinylated pUC 18 plasmid DNA control probe). For SIV ISH reactions, sections of lymph node from a SIVmac239-infected rhesus macaque served as both positive and negative control (when incubated with SIV antisense or sense probes, respectively). Additional negative controls included sections of normal rhesus kidney incubated with SV40 probe and sections of lymph node from a SIV-negative rhesus macaque incubated with SIV antisense probe.

## Results

### Plasma viral loads in monkeys infected with SHIV-1157i or passaged virus

**Figure 1:**
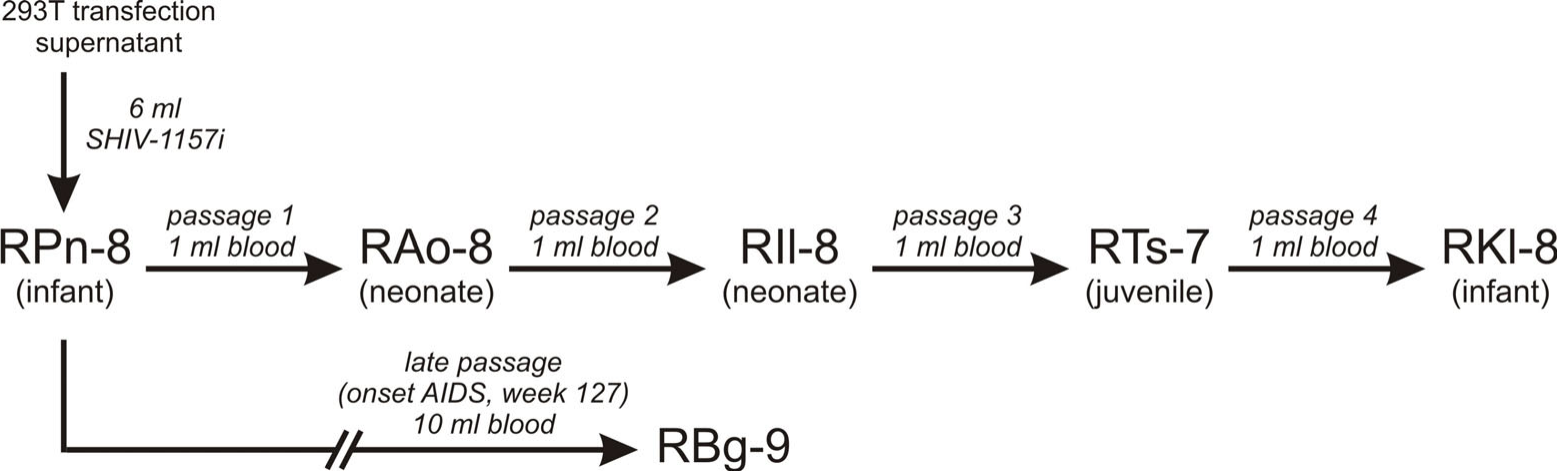
**Serial passage of SHIV-1157i in rhesus monkeys for viral adaptation**. The first animal was inoculated i.v. with cell-free supernatant from 293T cells transfected with the infectious molecular clone SHIV-1157i; subsequent animals were inoculated i.v. through serial blood transfer. The neonatal period comprises birth to one month; infancy the period up to one year, and juvenile monkeys are aged between one and five years.

**Figure 2:**
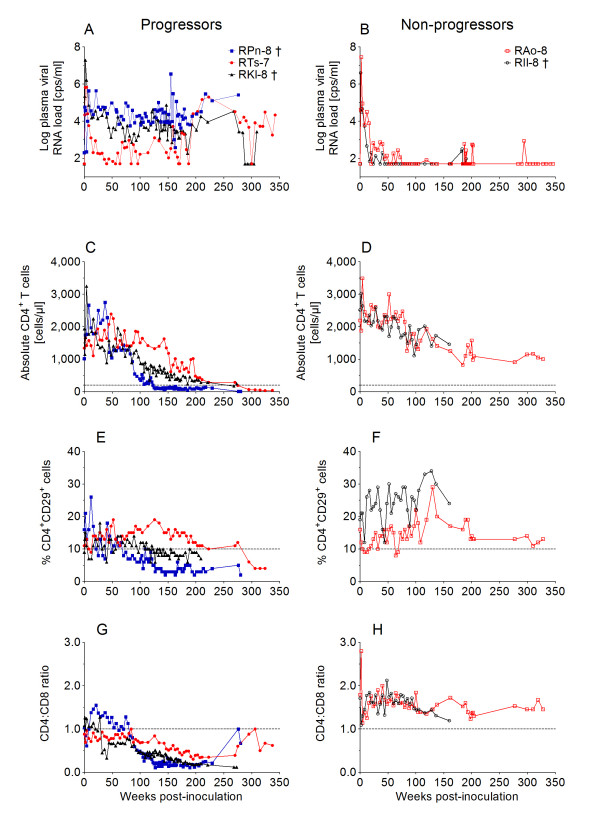
**Plasma vRNA loads and T-cell subsets in rhesus monkeys inoculated with SHIV-1157i or passaged virus**. The five animals used for virus adaptation were grouped into progressors and non-progressors. (A, B) Plasma vRNA loads. (C, D) Absolute CD4^+ ^T-cell counts. (E, F) Percentage CD4^+^CD29^+ ^memory T cells. (G, H) CD4:CD8 ratios. The dashed lines in panels C and D designate 200 cells/μl, the case definition threshold for human AIDS. In panels E and F, the dashed line at 10% indicates the lower limit of normal for the percentage of CD4^+^CD29^+ ^memory T cells. The threshold of detection of vRNA was 50 copies/ml. †, euthanasia due to AIDS-related disease (RPn-8) or unrelated reasons (RIl-8); monkey RKl-8 died during blood collection.

The details of the molecular cloning and biological characterization of SHIV-1157i have been previously published [8]. For the rapid animal-to-animal passage of SHIV-1157i, we used five rhesus monkeys (RM); the first animal was inoculated with 6 ml of cell-free virus obtained from 293T cells transfected with the infectious molecular clone, SHIV-1157i (Figure 1). At week 1 post-inoculation (p.i.), plasma viral RNA (vRNA) loads were measured and if found to be ≥ 10^4 ^copies/ml, 1 ml whole blood was transfused to the next animal a week later, the time point of the expected peak viremia (Figure 1). Plasma vRNA loads, absolute numbers of CD4^+ ^T cells, percentage CD4^+^CD29^+ ^memory T cells, and CD4:CD8 T-cell ratios were monitored longitudinally in peripheral blood in all RM. The five RM from the initial virus passage were divided into two groups: progressors (RPn-8, RTs-7, RKl-8) and non-progressors (RAo-8, RIl-8) (Figure 2). Both groups showed an initial peak of viremia within the first 2 weeks p.i. and seroconverted within 6 weeks. Compared to the first virus recipient, RPn-8, the four subsequent RM had peak vRNA loads that were 1–2 logs higher. After seroconversion, the progressors remained viremic with plasma vRNA levels ranging from 10^3 ^to 5 × 10^6 ^copies/ml, although plasma vRNA levels were occasionally undetectable in RTs-7 (Figure 2A). In contrast, the non-progressors controlled viremia after the initial high peak vRNA levels, and remained aviremic except for occasional blips that did not exceed 10^3 ^copies/ml (Figure 2B). Overall, the viral set points of the progressors were 1–4 logs higher compared to non-progressors; among progressors, only RTs-7 showed a relatively low vRNA level, with a setpoint of 10^4 ^copies or less/ml plasma throughout the latter portion of the observation period (Fig 2A).

### Pathogenicity of passaged virus

In all progressors, peripheral blood CD4^+ ^T-cell depletion occurred gradually, often first noted in the CD4^+^CD29^+ ^memory T-cell population (e.g., in RPn-8, Figure 2E). R5 viruses primarily infect and destroy memory CD4^+ ^T cells, a T-cell subset that expresses the CCR5 co-receptor [10]. The three progressor animals showed slow but persistent reductions in CD4^+ ^memory T cells (Figure 2E), whereas the non-progressors showed no such decline (Figure 2F). The massive loss of CD4^+ ^T cells that accompanies most untreated HIV infections results in a persistent inversion of the CD4:CD8 T-cell ratio, which serves as another important biomarker of lentiviral pathogenicity. In all progressors, CD4:CD8 T-cell ratios decreased below the normal pre-inoculation range of 0.7–1.4 for this group (Figure 2G). In contrast, there was no decrease in the CD4:CD8 ratios of non-progressors (Figure 2H).

All progressors developed AIDS as defined by persistent CD4^+ ^T-cell depletion below 200 cells/μl, the Centers for Disease Control (CDC)-established surveillance case definition threshold for human AIDS [11] (Figure 2C). The decrease in peripheral CD4^+ ^T cells observed in the two non-progressors is consistent with the normal age-related decline. Of note, both non-progressors (RAo-8 and RIl-8) were inoculated as neonates. Like human neonates, RM have CD4^+ ^T-cell counts in the range of 3000 – 4000 cells/μl at birth, which gradually decline to levels seen typically in adults (Figure 2D).

### Passage of late-stage virus

**Figure 3:**
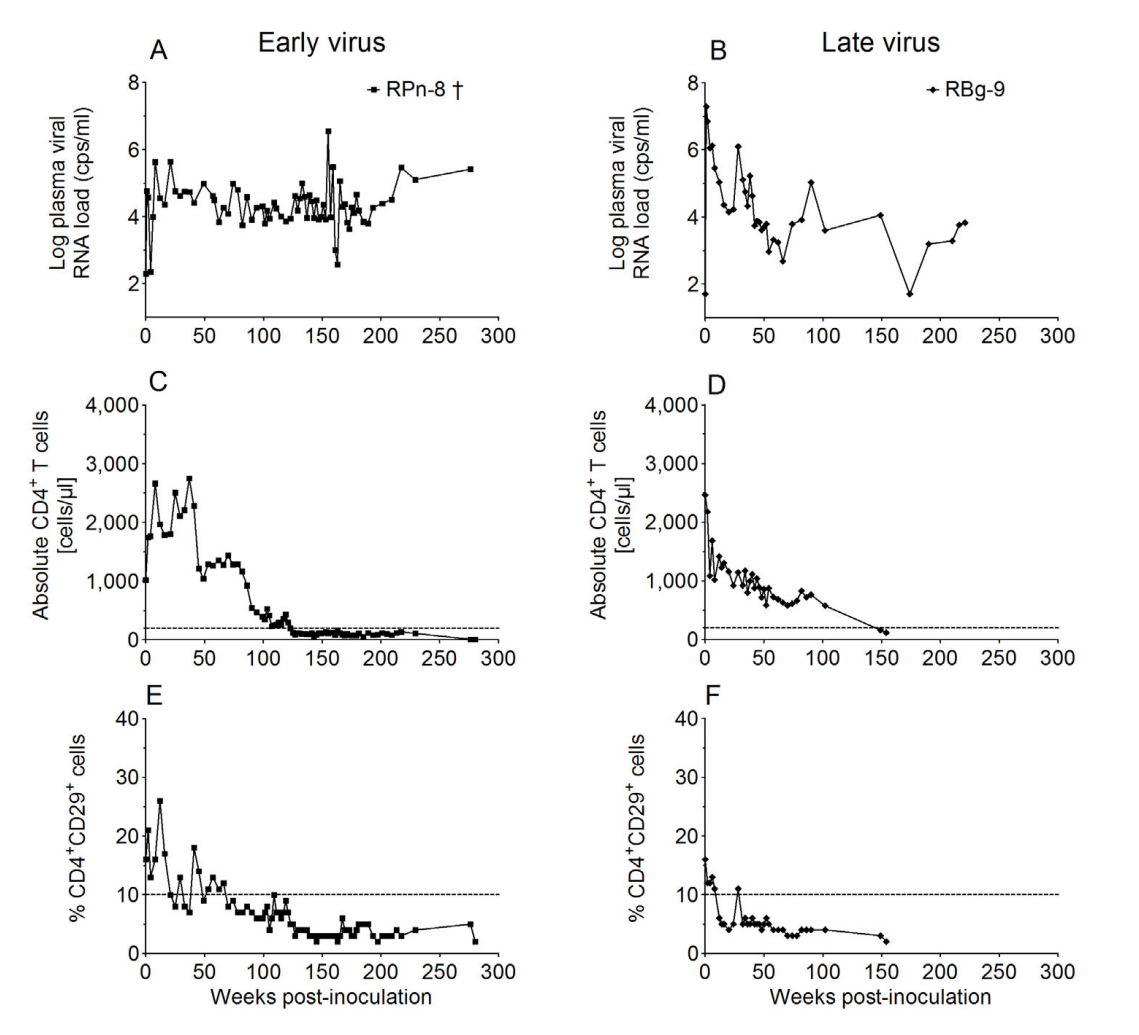
**Disease progression caused by the parental and late viruses**. Comparison of RPn-8 inoculated with SHIV-1157i and RBg-9 inoculated with the late virus (after AIDS had developed in monkey RPn-8). Panels show plasma vRNA loads (A, B), absolute CD4^+ ^T cells (C, D) and percentage CD4^+^CD29^+ ^memory T cells (E, F).

After monkey RPn-8, the first RM of the SHIV-1157i passage group, developed AIDS at week 123 p.i (Figure 2C), we sought to determine whether SHIV-1157i had acquired a more virulent phenotype *in vivo*. At week 127 p.i., 10 ml of whole blood was transfused from RPn-8 to naïve macaque RBg-9. Indeed, peak viremia in the recipient was approximately 2 logs higher than that induced by the parental infectious molecular clone in the donor, RPn-8 (Figures 3A and 3B; and [8]). RBg-9 also experienced a more rapid depletion of CD4^+^CD29^+ ^memory T cells in peripheral blood (week 12, Figure 3F) than RPn-8, and has progressed to AIDS.

### Virus-induced pathology

To determine the extent of disease induced by SHIV-1157i and passaged progeny virus, complete necropsies with histopathological evaluations were performed on the two monkeys (RPn-8 and RKl-8) lost to the complications of AIDS. Two other monkeys (RTs-7 and RBg-9) are alive with AIDS at the time of this writing.

RPn-8 consistently maintained fewer than 200 CD4^+ ^T cells for approximately three years, starting at week 123 p.i. RPn-8 developed intermittent diarrhea that progressed to watery diarrhea and became unresponsive to treatment, causing significant weight loss and ultimately requiring euthanasia at week 280 p.i. At the time of necropsy, RPn-8 had a CD4^+ ^T-cell count of 10 cells/μl.

RKl-8 had fewer than 200 CD4^+ ^T cells for almost one year before it died for unknown reasons during exam for acute onset of ataxia. At the time of death, the animal had a CD4^+ ^T-cell count of 232 cells/μl.

### SHIV-1157i-induced pathogenesis: histopathological evaluation

**Figure 4:**
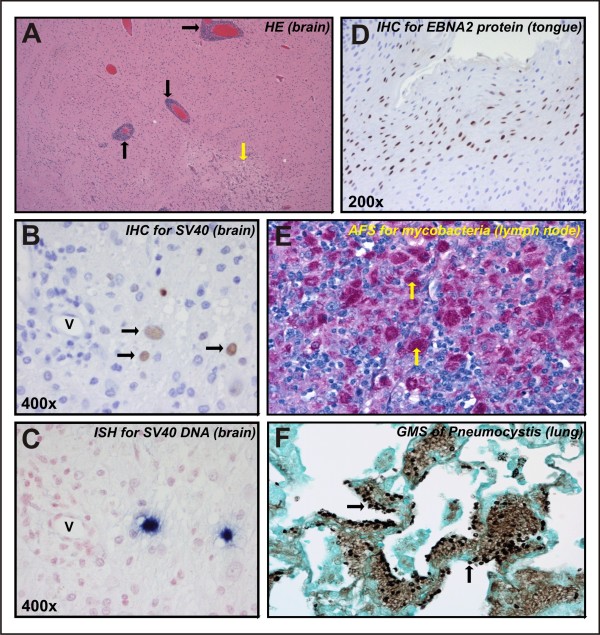
**Histological examination of RKl-8 (Panel A-C) and RPn-8 (Panel D-F)**. (A) Meningoencephalitis in RKl-8 brain, characterized by perivascular infiltrates ("perivascular cuffs") of mononuclear leukocytes (arrows) within the cerebral parenchyma, typical of viral encephalitis. Rarefaction of the white matter, consistent with demyelination, is also present (yellow arrow). (B, C) SV40 meningoencephalitis in RKl-8. SV40-positive cells were localized in the same regions by immunohistochemistry (IHC) (B) and in situ hybridization (ISH) (C). IHC for large T antigen shows SV40 positive cells (arrows) adjacent to a vessel (V) surrounded by inflammatory cells. SV40 DNA is localized within cells by ISH (blue NBT/BCIP chromogen) in a serial section of panel B. (D) Rhesus lymphocryptovirus infection. IHC for EBNA 2 on serial section of tongue shown in additional file 5A, demonstrating widespread localization of EBNA 2 protein expression in nuclei of mucosal epithelial cells (brown chromogen). (E) Mycobacterial infection in RPn-8. Acid fast stain of mesenteric lymph node reveals large numbers of mycobacteria-filled macrophages (magenta color; arrows). (F) Pneumocystis pneumonia in RPn-8. Section of lung stained by Gomori methenamine silver (GMS) technique to localize fungal organisms. *Pneumocystis *organisms (arrows) within the foamy exudate appear as crescent-shaped or folded spheres.

Histopathological evaluation of RPn-8 revealed disseminated mycobacteriosis, involving the small intestine, colon, liver, kidneys, lung, bone marrow, and mesenteric, peripancreatic and periaortic lymph nodes (additional file 1), which was confirmed by acid fast stain (Figure 4E). The presence of *Pneumocystis *spp. was noted in the lungs (additional file 2) and confirmed using Gomori methenamine silver stain (Figure 4F). Mycobacteriosis and Pneumocystis pneumonia are typical opportunistic infections in rhesus macaques with AIDS. Additional lesions in RPn-8 included focal candidiasis in the oral mucosa, and cryptosporidial tracheitis (additional file 3) and nasopharyngitis. Epstein Barr virus-like inclusions were observed in the mucosal epithelium of the tongue, and immunohistochemistry (IHC) for EBNA 2 provided a definitive diagnosis of rhesus lymphocryptovirus infection (Figure 4D and additional files 4 and 5).

The most prominent histopathological finding in RKl-8 was a multifocal meningoencephalitis attributed to SV40 infection, characterized by prominent mononuclear cell infiltrates surrounding venules in the meninges and extensive perivascular cuffing within the brain parenchyma (Figures 4A, additional file 6A). Other CNS findings included focal rarefaction of the cerebral white matter associated with inflammation (additional file 6B). In situ hybridization (ISH) for SIV gag and pol RNA failed to identify productively infected cells within inflammatory infiltrates, which suggested that the encephalitis was not a direct result of SHIV infection but rather was secondary to an opportunistic agent. Determination of proviral DNA load by PCR confirmed a low level of SHIV infection of the brain tissues (data not shown). Although viral inclusion bodies were not readily apparent, the presence of oligodendrocytes and astrocytes with swollen, euchromatic nuclei and occasional gemistocytic astrocytes within and surrounding the inflammatory lesions were suggestive of SV40 infection. IHC for SV40 large T antigen and ISH for SV40 DNA revealed the presence of large numbers of SV40-infected cells within encephalitic lesions and in the normal tissue surrounding lesions, providing confirmation of SV40 meningoencephalitis (Figure 4B, 4C and additional file 7).

Other histopathological findings in macaque RKl-8 included lentiviral arteriopathy, as evidenced by intimal thickening and fibrosis, luminal narrowing, occasional vasculitis, and rare thrombosis of the periaortic vasculature, as well as in medium and large arteries in the kidneys, colon (additional files 8 and 9), and lungs. In addition, there was follicular depletion and lymphoid atrophy of secondary lymphoid organs (spleen and lymph nodes), and cryptosporidial enteritis, confirmed by the presence of moderate numbers of cryptosporidial organisms within small intestinal crypts.

### Envelope evolution of SHIV-1157i

**Figure 5:**
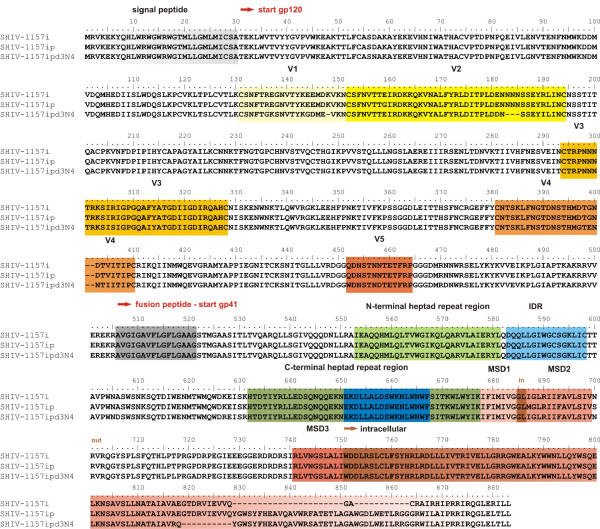
**Sequences analysis of SHIVs**. Alignment of Env amino acid sequences SHIV-1157i, SHIV-1157ip and SHIV-1157ipd3N4. Prominent domains of gp160 are highlighted in color and labeled. V1-V5 = variable loops V1-V5 gp120; IDR = immunodominant region gp41; MSD1-MSD3 = membrane spanning domains 1–3.

We analyzed sequences of the original virus clone (SHIV-1157i), the virus re-isolated week 6 p.i. from RKl-8 after passage through five rhesus monkeys (SHIV-1157ip), and the virus re-isolated from RPn-8 four weeks after the onset of disease (SHIV-1157ipd3N4). The data, partially published by Song *et al*. [8], show common amino acid substitutions in the variable loops of gp120 (V1-V4) for SHIV-1157ipd3N4 as well as an amino acid substitution for N295, which is part of the 2G12 epitope rendering this virus less sensitive for 2G12-mediated neutralization (Figure 5). Additionally, SHIV-1157ip and SHIV-1157ipd3N4 have an insertion in the intracellular part of gp41 (Figure 5).

## Discussion

Here we describe a SHIV which: 1) encodes *env *of a recently transmitted, pediatric HIV clade C strain from Zambia; 2) is highly replication competent as shown by long-term follow up of an initial cohort of macaques used to adapt the infectious molecular clone of this SHIV-C, SHIV-1157i; 3) uses R5 as coreceptor for viral entry [8]; and 4) is pathogenic with gradual disease progression to AIDS.

It is known that 90% of all HIV infections among humans occur through mucosal transmissions. SHIV-1157ip, the animal-passaged, biological isolate derived from the original SHIV-1157i, was shown to be mucosally transmissible. This isolate was used as oral challenge virus in a recent vaccine study [12], which was preceded by a formal titration through the oral route to determine the 50% animal infectious dose (AID_50_) of the SHIV-1157ip challenge stock.

The parental infectious molecular clone, SHIV-1157i, encodes *env *of a recently transmitted HIV-C. Our rapid animal-to-animal adaptation was designed to avoid nAb-mediated selection pressure by transferring virus at peak viremia from one donor to the next recipient. Since peak viremia occurs at two weeks p.i., nAbs will not yet have formed and thus, our adaptation strategy likely preserved the important structural characteristics of the recently transmitted HIV-C Env 1157i molecule. Interestingly, recently transmitted HIV-C was shown to be remarkably sensitive to neutralization in a study that prospectively followed HIV-discordant heterosexual couples [13]. Virus isolated from the newly infected partner was significantly more neutralization sensitive than contemporaneous virus isolated from the infected source person [13]. Moreover, the newly transmitted HIV-C gp120 molecules had significantly shorter amino acid lengths in the V1 to V4 region as well as fewer potential N-linked glycoprotein sites compared to the HIV-C quasispecies circulating in the source persons [13]. The V1 to V4 amino acid lengths in viruses of such newly HIV-C-infected Zambian individuals was also significantly shorter compared to virus from individuals with newly acquired HIV-B infection [14]. These data imply that recently transmitted HIV-C gp160 exist in a more open configuration and may expose neutralizing epitopes which become inaccessible during chronic infection. These special characteristics of recently transmitted HIV-C Env may have implications for anti-HIV-C vaccine design. Whether these observations hold for recently transmitted virus strains of other clades and for other transmission routes has been questioned [14-16]. Of note, however, shorter gp120 V1 to V2 amino acid lengths in recently transmitted HIV clade A (HIV-A) sequences in comparison to HIV-A sequences in the Los Alamos database were also reported [17]. Consequently, SHIV-1157ip with its recently transmitted HIV-C Env insert may turn out to be a valuable tool to assess vaccine efficacy in primate model studies.

Late stage SIV has been described as more virulent compared to early forms [18]. To test whether a similar increase in virulence would occur with SHIV-C, we performed a late blood transfer into monkey recipient RBg-9 after AIDS had developed in the first inoculated monkey, RPn-8, in which the late-stage virus, SHIV-1157ipd, had evolved during 127 weeks of continuous viremia. SHIV-1157ipd appeared to be more virulent than the early SHIV-C form by inducing higher peak vRNA loads and depleting the CD4^+^CD29^+ ^memory T-cell population in RBg-9 within a few weeks only. The full pathogenic potential of the late-stage virus was demonstrated by total CD4^+ ^T cells in RBg-9 dropping below 200 cells/μl blood. Notably, SHIV-1157ipd3N4 [8] was derived directly from this late-stage biological isolate SHIV-1157ipd; the infectious molecular clone SHIV-1157ipd3N4 was engineered to encode additional NF-κB sites in the LTRs to increase replicative capacity. SHIV-1157ipd3N4 retained its R5 tropism, is mucosally transmissible, is pathogenic and causes AIDS, and has also already been used in vaccine studies by our group ([12], unpublished data).

Although SHIV-1157ip is not the first non-clade B R5 SHIV [19-22], SHIV-1157i and its progeny have certain features which are not shared by other chimeras. None of the previously described non-clade B SHIVs was shown to be mucosally transmissible and to cause AIDS in rhesus macaques. Of note, some of the non-clade B chimeras use CXCR4 as coreceptor or are dual tropic (reviewed in [23]).

We and others [23,24] have suggested that primate models of HIV infections should not only reflect key aspects of HIV transmission among humans, but also mirror the target cell specificity during acute infection and the natural, gradual disease progression seen in HIV-infected humans. Key findings of Nishimura et al. [25] showed that acute infections of R5 and X4 viruses differ in targeting separate CD4^+ ^T-cell subsets, resulting in distinct patterns of subsequent CD4^+ ^T-cell depletion. R5-tropic SIV_mac239 _or SIV_smE543 _strains preferentially target and destroy CCR5^+ ^memory CD4^+ ^T cells. After acute viremia, SIV-infected monkeys progressed to AIDS over several months and showed selective depletion of memory cells with a complete loss at time of death. In contrast, SHIV_DH12R _or SHIV_KU1 _use CXCR4 for infection, which is preferentially expressed on naïve CD4^+ ^T cells. SHIV89.6P, one of the most widely used strains in monkey models, is dual tropic in vitro but acts like an X4 virus *in vivo *[26]. SHIV89.6P as well as X4 SHIV strains induce massive elimination of naïve CD4^+ ^T cells, leading to a rapid and mostly irreversible loss of peripheral blood CD4^+ ^T cells within approximately two weeks post-inoculation. This acute onset of severe T-cell depletion, which is also seen with the related SHIV89.6PD [27], does not reflect the clinical course of HIV infection seen in humans. In contrast, the gradual disease progression caused by our R5-tropic SHIV-1157i-derived viruses is more reflective of HIV disease progression in humans.

Several R5-tropic SHIV-B strains have been described [28-30] based upon HIV_SF162 _or HIV_Ba-L _*env *inserts, giving rise to SHIV_SF162P3_/SHIV_SF162P4 _and SHIV_Ba-L _[28,29]. SHIV_SF162P3 _and SHIV_SF162P4 _differ in their monkey passage histories and in their neutralization sensitivities, with P4 classified as Tier 1 virus and P3 as a more difficult to neutralize Tier 2 strain (David Montefiori, personal communication). SHIV_SF162P3 _induces gradual CD4+ T-cell loss and causes AIDS in some but not all rhesus macaques [31]. Recently, Pahar et al. [32] using vaginal SHIV_SF162P3 _challenge observed control of viremia with modest depletion of the memory CD4^+ ^T cell subset; however, these animals were followed only until day 84 p.i. Overall, SHIV_SF162P3 _induced progressive disease leading to AIDS in 6 out of 11 rhesus monkeys with systemic infection after intravaginal challenge [33]; the time to development of AIDS varied from 5.5 weeks to 104 weeks p.i SHIV_SF162P3 _was adapted to rhesus monkeys cumulatively over a time span of 26 weeks. In contrast, the animals described in our cohort were infected with an R5 SHIV-C that was in the process of being adapted. Even virus reisolated from the last recipient in the serial transfer, monkey RKl-8, replicated only a total of 14 weeks in rhesus macaques. Not surprisingly therefore, SHIV-1157i (the parental virus in monkey RPn-8) and its progeny induced progression to AIDS that was somewhat slower compared to SHIV_SF162P3_. Also a consideration is the relatively small numbers of animals followed long-term with systemic SHIV_SF162P3 _or SHIV-1157ip infection. Nevertheless, the overall biological properties of the two R5 SHIVs seem similar in outbred rhesus monkeys, with mucosal transmissibility and gradual disease progression the key features. SHIV_SF162P3 _has also successfully been used in a monkey model for mother-to-child transmission to evaluate key parameters in perinatal HIV transmission [34,35].

The related Tier 1 virus, SHIV_SF162P4_, was used as a challenge virus in a recent vaccine study using HIV-1 SF162 Env as immunogen; the results showed that antibodies induced by the homologous vaccine could protect rhesus macaques from intravaginal challenge [36]. R5-tropic SHIV_Ba-L _was used in only two short-term vaccine efficacy studies using intrarectal challenge [37,38]. No information has been published as yet regarding the pathogenicity of SHIV_Ba-L_, whereas SHIV_SF162P3 _and SHIV_SF162P4 _are known to cause progressive disease, including AIDS in a gradual downhill course.

Recently, the group of Cecilia Cheng-Mayer described for the first time a coreceptor switch of an R5 SHIV-B, SHIV_SF162P3N _[39-41]. Coreceptor switch from CCR5 to CXCR4 is observed in approximately 50% of HIV-B-infected humans but only rarely in HIV-C-infected individuals [39,41,42]. The increase in X4 variants is associated with rapid CD4^+ ^T-cell loss and progressive disease [3,43-45]. To reflect HIV transmission and assess AIDS vaccine efficacy, it is important to examine the underlying biology for this coreceptor switch. Since this phenomenon is rare among HIV-C, it will be interesting to test whether any of our R5 SHIV-C strains have the potential for coreceptor switch in future studies.

## Conclusion

Our long-term follow up of animals infected with SHIV-1157i and variants thereof document for the first time the pathogenicity of a R5 clade C SHIV with gradual disease progression to AIDS manifested by opportunistic infections typically seen in HIV infections in humans. This suggests that these viruses are biologically relevant tools to evaluate the efficacy of candidate anti-HIV-C vaccines in nonhuman primates.

## Competing interests

The authors declare that they have no competing interests.

## Authors' contributions

RS, RAR and RMR designed the study. RAR, RS and HO performed experiments; JGE, ES and FJN coordinated and performed the primate studies. PS and SON performed pathological and histopathological analyses. ALC, VGK and NBS performed viral load measurements. MH, RAR, RS collected and analyzed data. MH, RAR, PS, SPO, RMR wrote the manuscript. All authors read and approved the manuscript.

## Supplementary Material

Additional file 1 Mycobacteriosis in RPn-8. Histopathological examination of mesenteric lymph node. The lymph node parenchyma is effaced with large numbers of epitheloid macrophages (arrows). Click here for file

Additional file 2 Pneumocystis pneumonia in RPn-8. The pulmonary alveoli are filled with a foamy exudate (arrows). Click here for file

Additional file 3 HE of trachea of RPn-8. Cryptosporidial organisms (arrows) on the luminal surface of the tracheal mucosa. Click here for file

Additional file 4 Lymphocryptovirus infection of the tongue of RPn-8. Epstein Barr virus-like inclusions (arrows) in the epithelium. Click here for file

Additional file 5 Immunohistochemistry for diagnosis of rhesus lymphocryptovirus infection. (A) Section of tongue from rhesus macaque RPn-8, shown at low magnification (200×) after staining with hematoxylin and eosin (H&E). (B) Higher magnification of A (400×), showing EBV-like intranuclear inclusions (arrows). (C) Higher magnification of Figure 4D (400×), showing intranuclear localization of EBNA 2 expression. Click here for file

Additional file 6 HE of the brain of RKl-8. Detailed pictures from Figure 4A. (A) Meningoencephalitis (20×). (B) Rarefaction (arrows) of the cerebral white matter (20×). Click here for file

Additional file 7 Diagnosis of SV40 meningoencephalitis in RKl-8. (A-C) IHC for SV40 large T antigen, revealing swollen, immunoreactive glial nuclei (brown chromogen) within encephalitic regions (A; with inflammatory cell infiltrate indicated by arrow) or in normal brain parenchyma (B and C) adjacent to areas of inflammation. (D) Lower magnification view showing a single SV40 positive cell by ISH (asterisk) adjacent to a perivascular cuff of inflammatory cells (arrow) within a region of inflammation and demyelination. Click here for file

Additional file 8 HE of colon (A) and kidney (B) of RKl-8. (A) Arteriopathy marked by intimal thickening and fibrosis. (B) Vascular changes in renal parenchyma. Click here for file

Additional file 9 HE of RKl-8. A recannalized thrombus in a blood vessel in the mesentery of the colon. Click here for file
