# Supplementary figures and images for: SHIV-1157i and passaged progeny viruses encoding R5 HIV-1 clade C env cause AIDS in rhesus monkeys
Source: Retrovirology. 2008 Oct 17;5:94. doi: 10.1186/1742-4690-5-94 (PMC2576354; doi:10.1186/1742-4690-5-94)

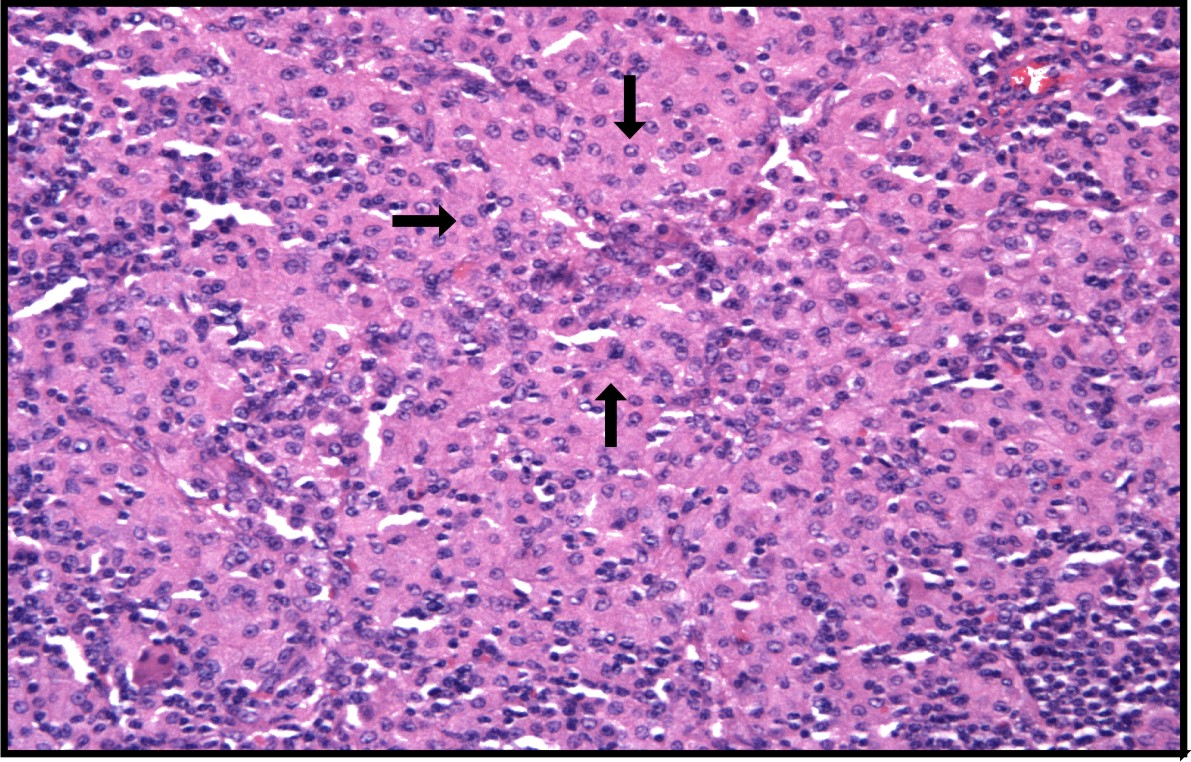

Supplement: Additional file 1 — Mycobacteriosis in RPn-8. Histopathological examination of mesenteric lymph node. The lymph node parenchyma is effaced with large numbers of epitheloid macrophages (arrows). [file 1742-4690-5-94-S1.jpeg]

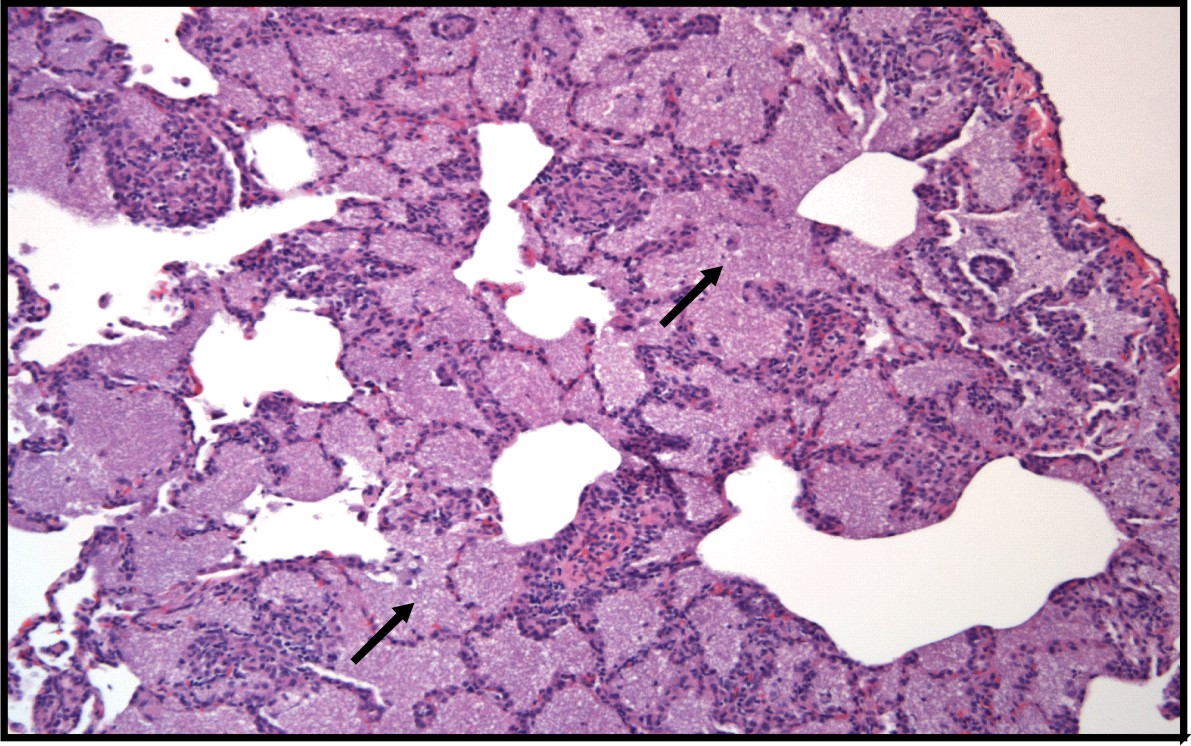

Supplement: Additional file 2 — Pneumocystis pneumonia in RPn-8. The pulmonary alveoli are filled with a foamy exudate (arrows). [file 1742-4690-5-94-S2.jpeg]

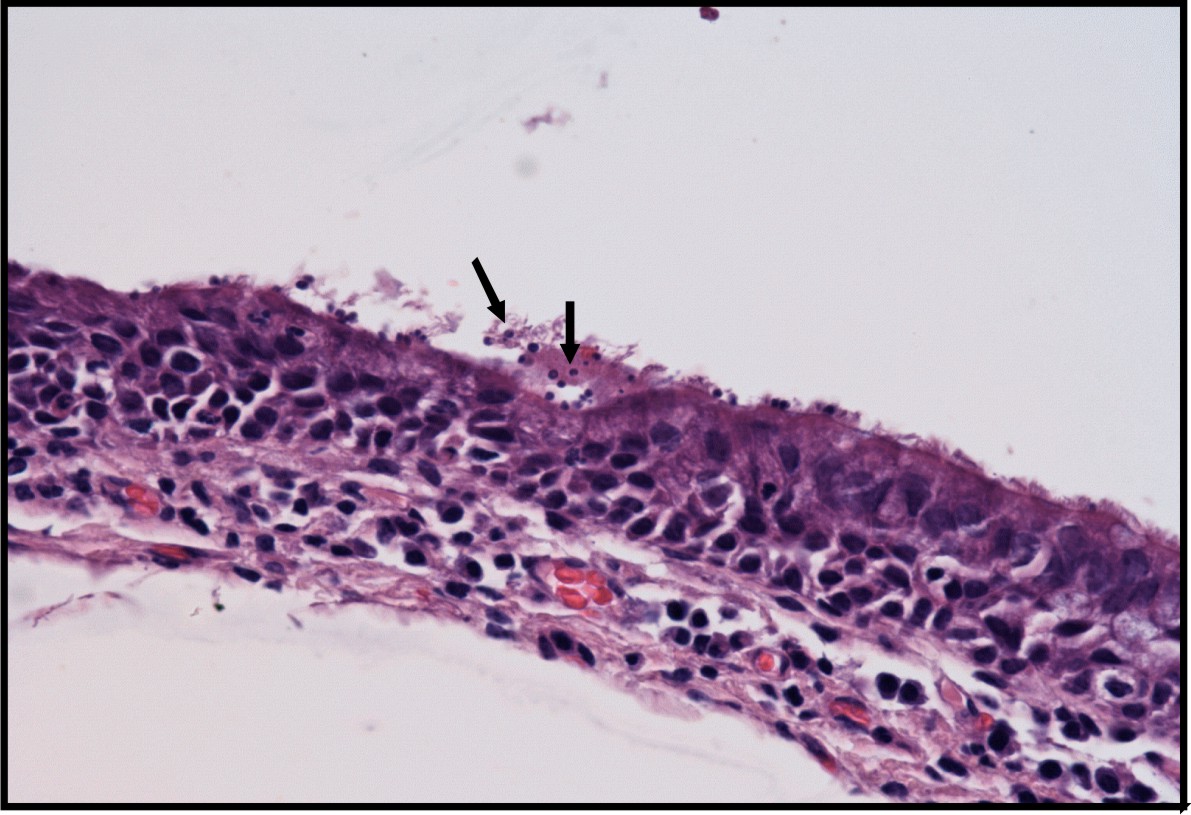

Supplement: Additional file 3 — HE of trachea of RPn-8. Cryptosporidial organisms (arrows) on the luminal surface of the tracheal mucosa. [file 1742-4690-5-94-S3.jpeg]

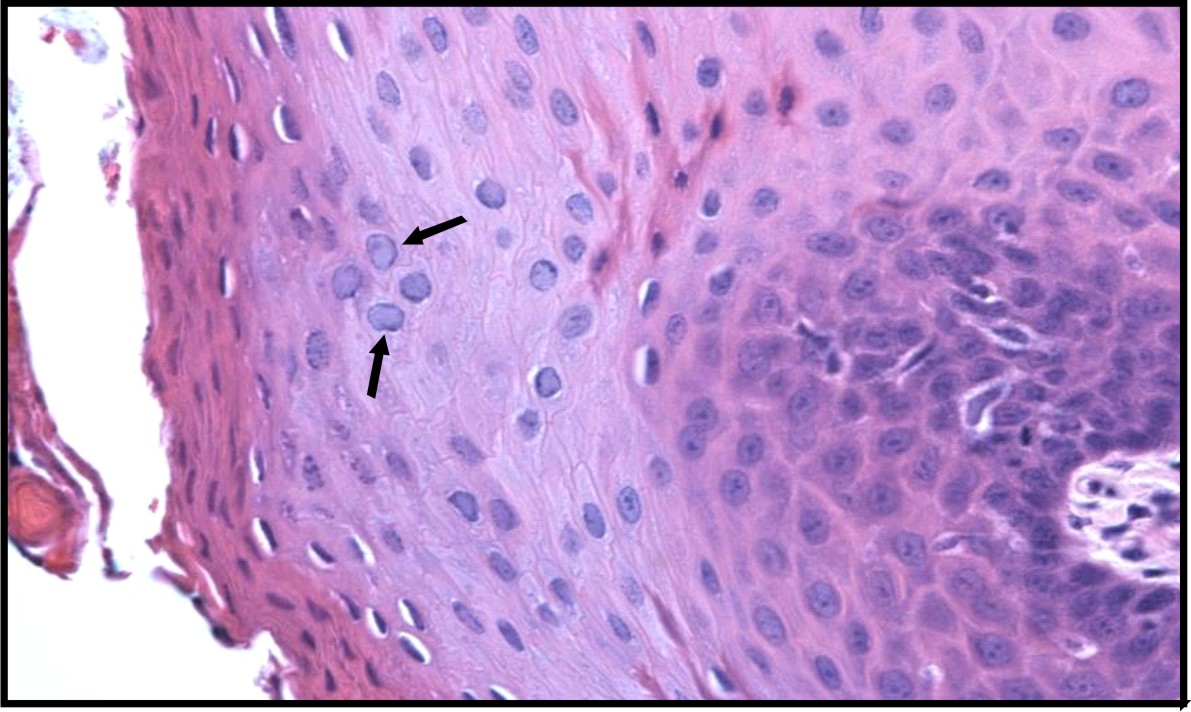

Supplement: Additional file 4 — Lymphocryptovirus infection of the tongue of RPn-8. Epstein Barr virus-like inclusions (arrows) in the epithelium. [file 1742-4690-5-94-S4.jpeg]

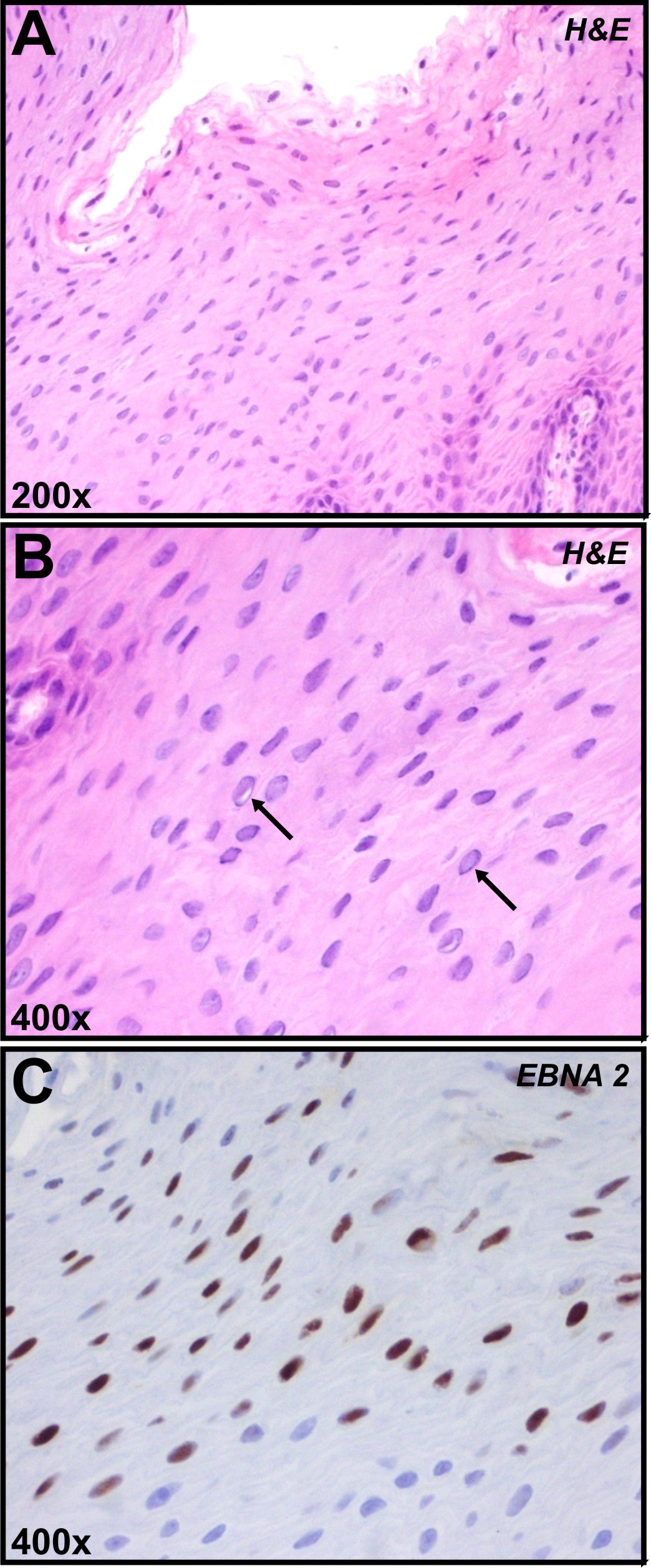

Supplement: Additional file 5 — Immunohistochemistry for diagnosis of rhesus lymphocryptovirus infection. (A) Section of tongue from rhesus macaque RPn-8, shown at low magnification (200×) after staining with hematoxylin and eosin (H&E). (B) Higher magnification of A (400×), showing EBV-like intranuclear inclusions (arrows). (C) Higher magnification of Figure 4D (400×), showing intranuclear localization of EBNA 2 expression. [file 1742-4690-5-94-S5.jpeg]

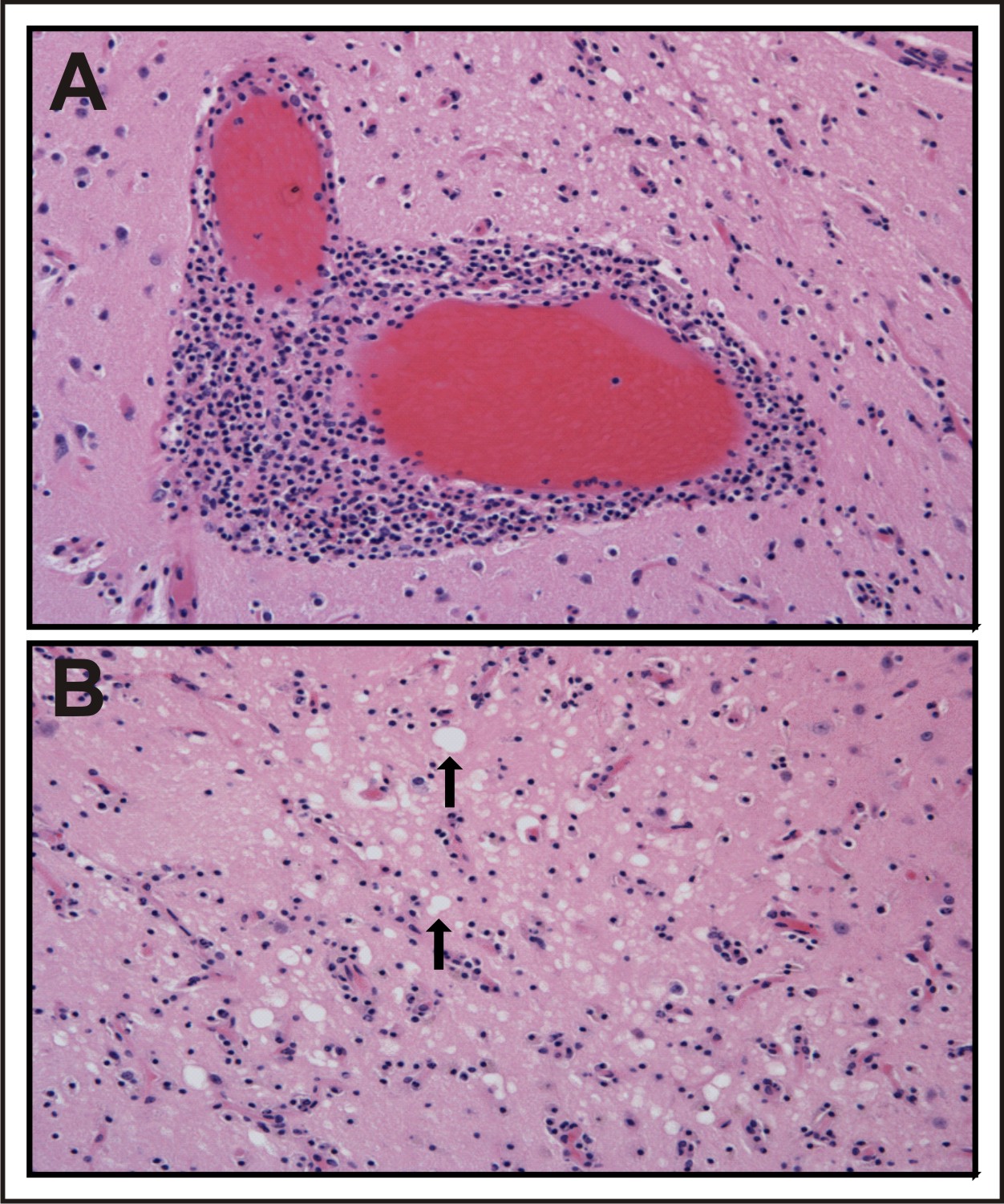

Supplement: Additional file 6 — HE of the brain of RKl-8. Detailed pictures from Figure 4A. (A) Meningoencephalitis (20×). (B) Rarefaction (arrows) of the cerebral white matter (20×). [file 1742-4690-5-94-S6.jpeg]

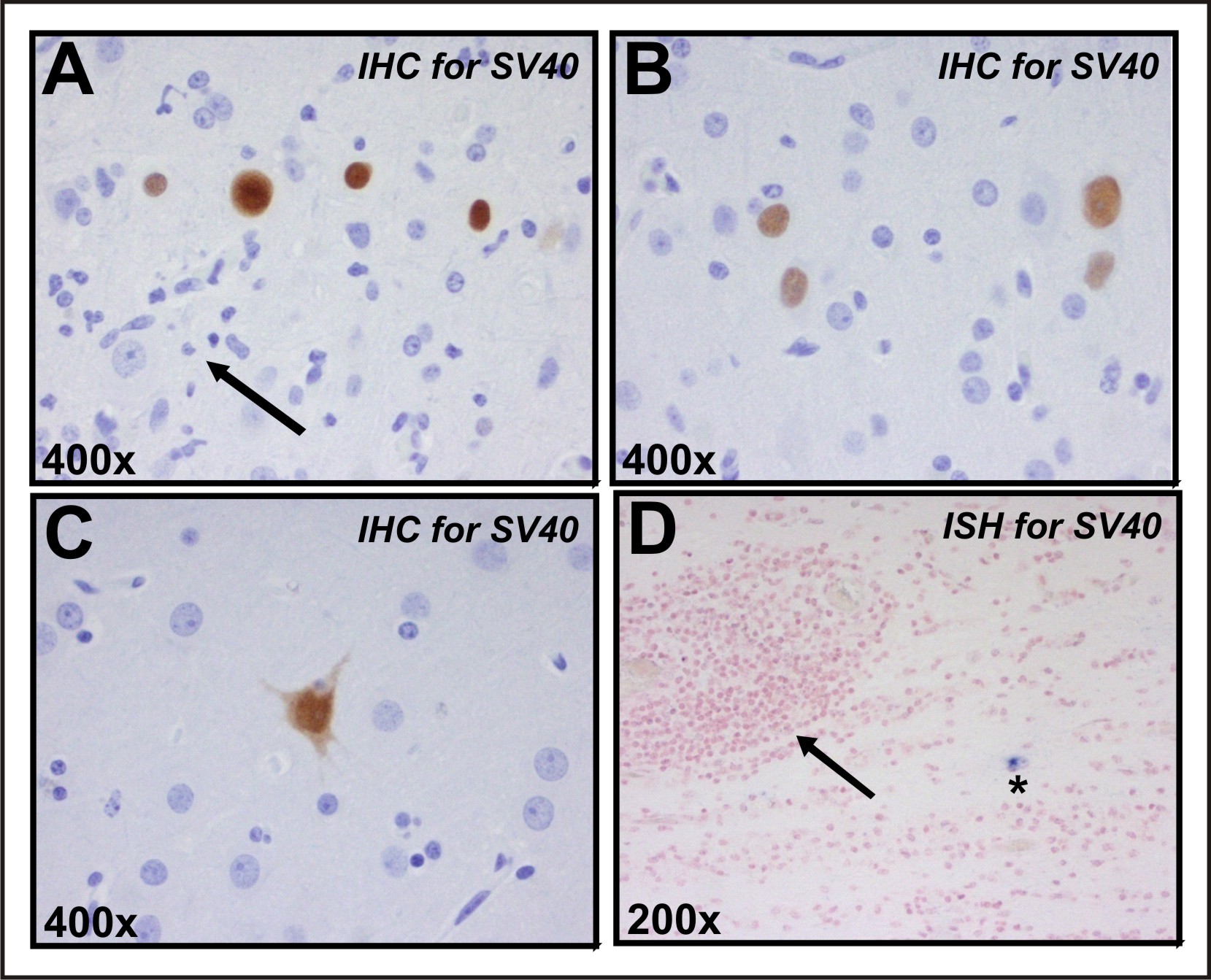

Supplement: Additional file 7 — Diagnosis of SV40 meningoencephalitis in RKl-8. (A-C) IHC for SV40 large T antigen, revealing swollen, immunoreactive glial nuclei (brown chromogen) within encephalitic regions (A; with inflammatory cell infiltrate indicated by arrow) or in normal brain parenchyma (B and C) adjacent to areas of inflammation. (D) Lower magnification view showing a single SV40 positive cell by ISH (asterisk) adjacent to a perivascular cuff of inflammatory cells (arrow) within a region of inflammation and demyelination. [file 1742-4690-5-94-S7.jpeg]

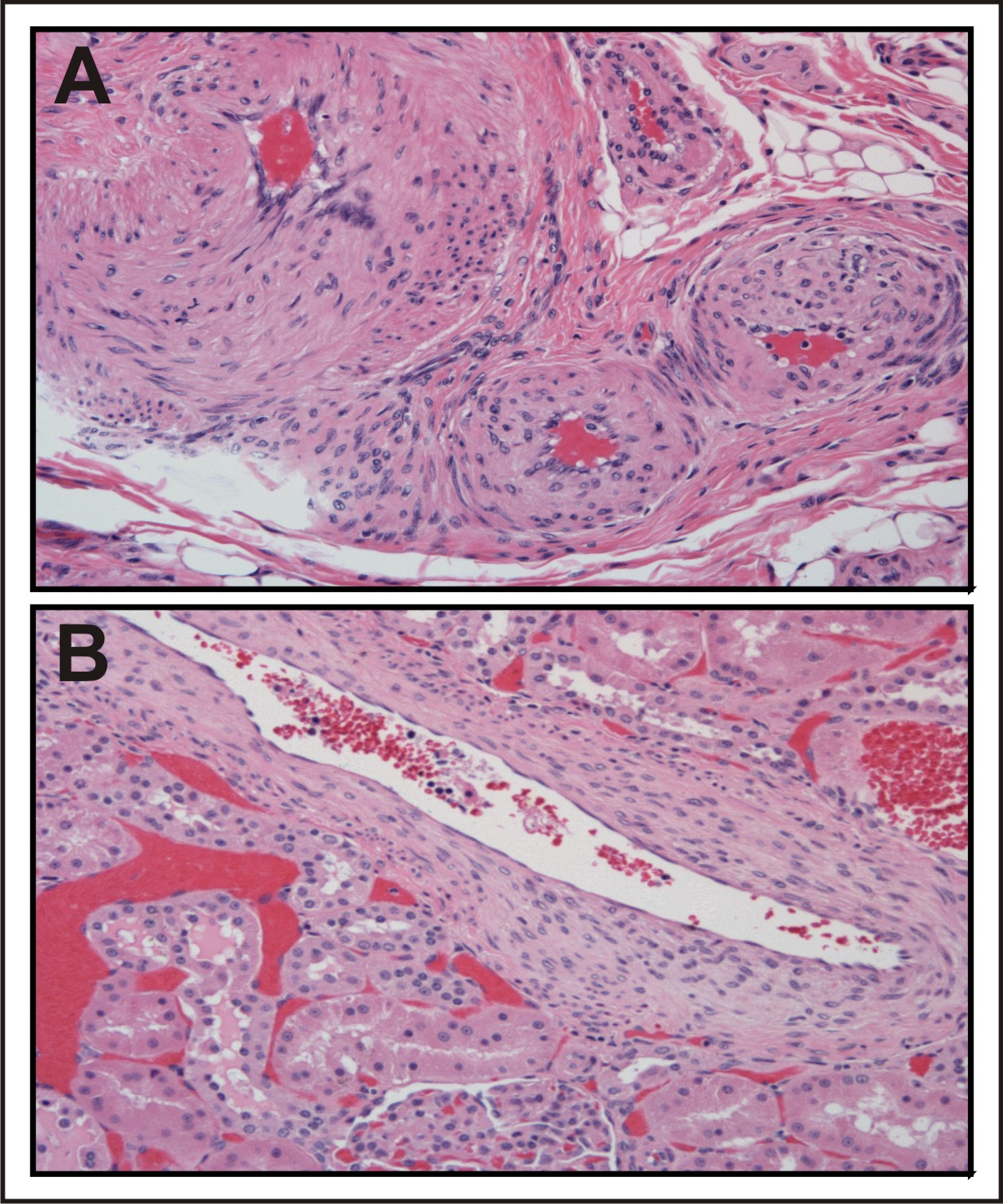

Supplement: Additional file 8 — HE of colon (A) and kidney (B) of RKl-8. (A) Arteriopathy marked by intimal thickening and fibrosis. (B) Vascular changes in renal parenchyma. [file 1742-4690-5-94-S8.jpeg]

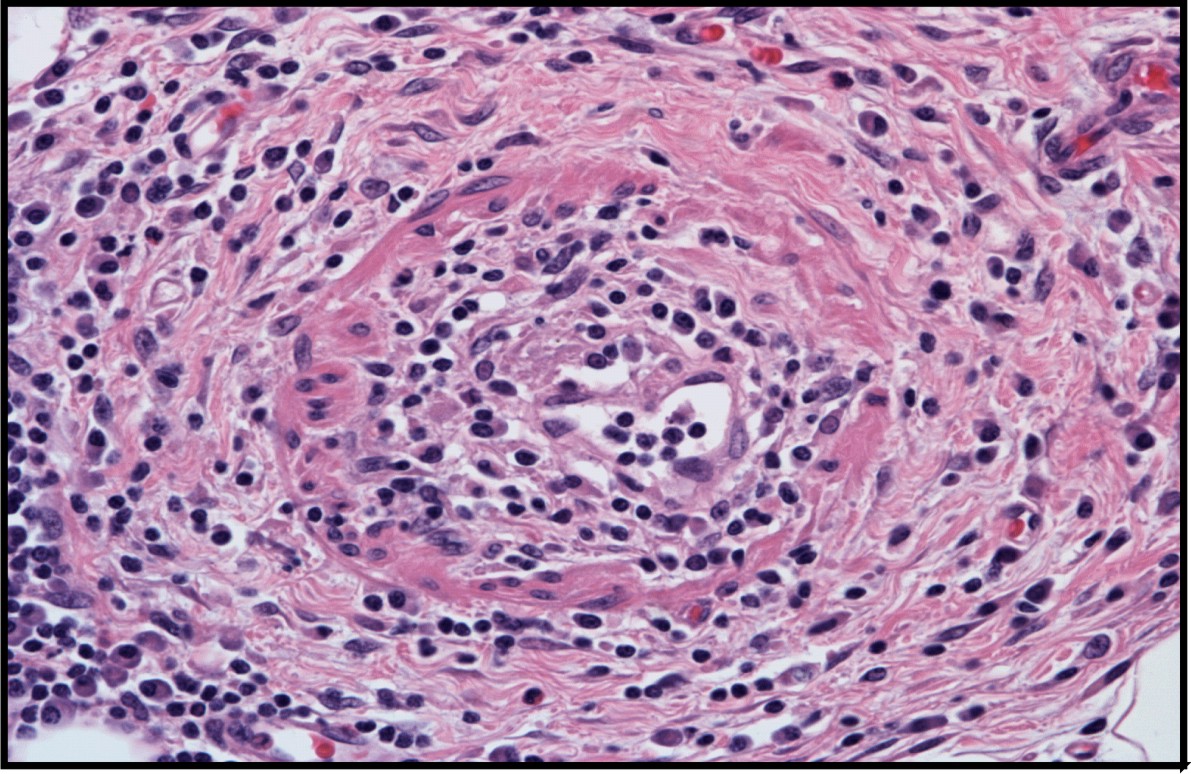

Supplement: Additional file 9 — HE of RKl-8. A recannalized thrombus in a blood vessel in the mesentery of the colon. [file 1742-4690-5-94-S9.jpeg]
